# Commentary: Physical time within human time

**DOI:** 10.3389/fpsyg.2023.1096280

**Published:** 2023-05-04

**Authors:** Michael Silberstein

**Affiliations:** Department of Philosophy, Elizabethtown College, Elizabethtown, PA, United States

**Keywords:** time, consciousness, relativity, cognitive neuroscience, IGUS

Why do we experience Presence, Passage, and Direction when none of these things is given to us in fundamental physics, i.e., in relativity? As Callender (2017, p. 27) puts it, “Peering into physical time is illuminating, but no amount of focusing will bring manifest time into view. It's not there.” While special relativity (SR) is said by some to yield a block universe, when we get to general relativity (GR) and many accounts of quantum gravity (QG), things become even worse due to these three phenomenological aspects of time being absent (Silberstein et al., 2018, chps. 3, 6, 7, 8; Huggett et al., 2013, p. 250). This is especially troubling for some as these three temporal features of conscious experience are the most essential and fundamental aspects of daily conscious life and the features of experience that foundational physics is most concerned about explaining (Smolin, 2019). As Callender (2017, p. 27) says, “physics is really the only science we have that explicitly takes *time itself* as one of its targets of study”.

It is important to begin by distinguishing two types of questions. There are physical/metaphysical questions, and there are phenomenological or experiential questions.

Traditionally, starting with Husserl, in a neo-Kantian transcendental spirit, the discipline of phenomenology has sought to “bracket” questions of experience from metaphysical questions. There are those who believe, however, that the metaphysical and physical features of time explain the phenomenological ones.

According to Price (2011, p. 277), the physical/metaphysical questions are as follows:

1. Is the Present moment objectively distinguished such that it is a frame-or-perspective-independent fact about which events are present as opposed to past or future?
2. Does time have an objective Direction such that for all events (e.g., two non-simultaneous events) the answer to which one is the earlier and which one is later, is a frame-or-perspective-independent fact? That is, for all events is there an objective fact about which Direction is toward the past, such as allegedly the Big Bang, and which toward the future, such as allegedly the heat death of the universe?
3. Irrespective of conscious observers, their frame of reference, or perspectives in the universe, is it an objective fact that there is a Passage or flow of time as suggested, for example, by dynamical presentism (i.e., there is an objective present, and it objectively moves/passes from past to future)?

Assuming SR and GR are true and complete and that our best theory of QG turns out to lack these three features of time as well, then the answer to each of these questions is “no.” This is because the relativity of simultaneity is said by many to strongly suggest “eternalism” or the “block universe”: the equal reality of the past, present, and future. Eternalism follows from special relativity precisely because there will be relativistic reference frames whose observers disagree about the temporal ordering of (spacelike separated) events into past, present, and future. That is to say, there will be disagreements as to which events are simultaneous with which. There will be frames of reference (such as planets at great distances from Earth or a spaceship moving by Earth at a large fraction of the speed of light), whose observers will disagree about how to order events in the universe into NOW-slices.

To use a film analogy, in the actual world, not everyone is watching the same movie. To spell it out, this suggests a block universe because if there are events such as a particular supernova explosion that is experienced by two different observers, but they do not agree as to when that event happened, the event must just be “there” statically, timelessly, to be experientable from both these different spatiotemporal perspectives. In principle, this will hold true for all events in spacetime. It is only from the “ant's-eye” perspective if you will that dynamical presentism seems like the best bet. From the 4D “God's-eye” perspective, as Hermann Weyl puts it, “The objective world simply *is*; it does not *happen*.” This is why the alien with the God's-eye perspective in Kurt Vonnegut's novel *Slaughterhouse Five* says the following: “I am a Tralfamadorian, seeing all time as you might see a stretch of the Rocky Mountains. All time is all time. It does not change. It does not lend itself to warnings or explanations. It simply is.” For a straightforward and streamlined argument for eternalism based on special relativity and the relativity of simultaneity, plus a few innocuous assumptions about the meaning of the word “real” (see Silberstein et al., 2018, chp. 2).

The implication of all this is that relativity (physics) is in no position to help explain any phenomenological features of temporal experience by offering an objective Present, objective Passage, or objective Direction; that is, we now have a mystery as to why we do not all experience the universe as the Tralfamadorians do.

As we will see, some might invoke cognitive neuroscience to dispel the mystery. But before we get there, it is important to note that there are moves one can make regarding physics and metaphysics. First, note that eternalism simply asserts the equal reality of the past, present, and future. Using the resources of Minkowski spacetime (M4), one is still free to try and cook up accounts of the Present, Passage, or Direction, however non-objective they may be. But such an account must explain the experience of these three features of time without the resources of say dynamical presentism such as modeled by Newtonian mechanics.

Second, one is free to deny that relativity is true and complete and many do. Smolin (2013, 2021) famously wants to add something like Passage, Presence, and Direction into his fundamental theory of physics. Smolin thinks that change, potentia, and the openness of the future are built into fundamental physics. Some interpretations of quantum mechanics, such as Bohmian mechanics, suggest the need for the addition of a preferred frame to relativity. Finally, many metaphysicians of time would say that the physics of time underdetermines the metaphysical nature of time—maybe there is more to the world than physics or even cognitive neuroscience.

However, neither Buonomano nor Rovelli seems to deny realism about Minkowski spacetime (M4) and neither seeks to supplement it with a preferred frame, etc. Both seem to acknowledge that given M4 alone, there is no preferred universal or global present, but at best many local presents. Simply put, they agree there is no unique way to carve 4D spacetime into individual 3D distributions of coexisting objects and events in order to create the individual frames for an objective film shared by all; that is, both seem to acknowledge that according to the relativity of simultaneity and the light postulate, there will be inertial reference frames that disagree about the ordering of events into the past, present, and future. Neither Buonomano nor Rovelli seems to want to take either of these two ways out.

Rovelli (2018, p. 209–110) and Buonomano (herein) are both on record as rejecting presentism and eternalism, but it is not clear what their physical or metaphysical alternative is. What is clear is that both want to resist saying that “time is an illusion, the world is static, and there is no change.” This is indeed a frequent claim made by eternalists and blockheads. For one attempt to reconcile eternalism and the phenomenology of time, see our book *Beyond the Dynamical Universe* (Silberstein et al., 2018).

If one does not take either of these two ways out, then it would seem an explanation of temporal experience must be grounded in cognitive neuroscience as Callender (2017) and Gruber et al. (2020, 2022) attempt to do. If one is committed to physicalism, ontological reductionism, mechanistic explanation, etc., there is no other option.

On the phenomenological side, what we want to explain is the experiential arrow of time which has the following features:

- Passage: the world is in constant flux such that the future becomes the present and the present becomes the past.
- Presence: the present moment is experienced as special or ontologically privileged.
- Direction: time appears to flow from a distinguishable past to a distinguishable future.

If one does not take one of the preceding two ways out, must the explanation for the phenomenology of time be exclusively the purview of psychology and cognitive neuroscience? The answer to this question depends on to what degree you think physics constrains or contradicts the phenomenology of temporal experience. In other words, how decoupled is the phenomenology of time perception from physics? While there may be no necessary contradiction between physics and cognitive neuroscience in this regard, as we have seen, it does seem that physics lacks objective Passage, Presence, and Direction, as none of these are present in relativity theory, our best theory of time. Are there nonetheless resources in relativity theory that can help explain temporal experience?

Callender (2017, p. 31), for example, takes the following view, “I think we can explain why manifest time arises for us in a world governed by our physical laws. But doing so, if I am right, will require embedding a subject like us in a world like ours, and not simply finding some structures in physics that plays[sic] the ‘manifest time' role”. Callender (2017, p. 306) is clear that he thinks physics provides some necessary conditions for explaining temporal experience but is non-sufficient. One of Callender (2017, p. 263)'s criticisms of a Smolin-type approach is that no matter what one does to modify a physical theory to give it something like Passage, Presence, and Direction, one must show that those modifications are the explainer/the cause of temporal experience. Part of Callender's point is that it seems absurd to think that we have special unknown sensory apparatus for detecting the physical esoterica of Passage and Presence.

Rovelli and Buonomano, on the other hand, seem to want to find a way of explaining temporal experience without either modifying our physical theories to include Passage, Presence, and Direction or relying *completely* on cognitive neuroscience for the answer. Rovelli (herein) suggests that the second law of thermodynamics underwrites Passage and Direction, but many of us have argued that thermodynamics and the second law in fact presuppose Passage and Direction, that is, time (Silberstein et al., 2018, p. 367–368). Regarding physics, Buonomano consoles himself as follows: “there is no empirical evidence to support a critical tenet of the block universe: that the past and future, physically speaking, are as real as the present.” This however is not a very powerful argument for ignoring relativity, because we have good theoretical and formal evidence in our physical models for many things we cannot now confirm empirically. As mentioned earlier, the well-confirmed relativity of simultaneity plus a few widely held assumptions indicate a block universe. Buonomano does not appear to reject realism about M4 or any of the other assumptions in question leading to a block universe.

Buonomano also suggests that the possibility of time travel would be a necessary and sufficient condition for eternalism. He also asserts that time travel is impossible (at least via closed-time-like curves which GR does allow in principle if not in practice). The problem here is that it is now widely accepted that presentism (the metaphysical view of time that only the present is real) is also consistent with the possibility of time travel (Effingham, 2020). In short, the possibility of time travel is a red herring when it comes to the presentism-vs.-eternalism debate. Of course, there is unlikely to be any crucial experiment that settles this debate.

My conclusion is that in this particular exchange, neither Rovelli nor Buonomano engages deeply with the best physical and metaphysical arguments for eternalism, but they may do so elsewhere. I also conclude that neither of them finds an alternative to modifying relativity, etc., or falling back on cognitive neuroscience. To see such an alternative, read our book.

Physics aside, both authors seem to agree that it is at least *partly* the job of cognitive neuroscience to explain the phenomenology of Passage, Presence, and Direction. Of course, they both acknowledge that changing certain physical facts, such as the metric signature of M4, would affect our temporal experience (Callender, 2017, p. 156).

Here is where GBM (herein) enters the story with the IGUS model that might do the trick of explaining the experiential arrow of time. The IGUS model is a computational, functionalist model that could be implemented by the brain to produce the experience of Passage, Presence, and Direction (Hartle, 2005). Whether or not there is any evidence that human brains do implement IGUS, I have no idea. I will assume the reader is familiar with the IGUS model and its many improvements as suggested by Callender (2017, p. 232–235, 247–261). Hartle says we should “build this robot,” and he believes that if done thoroughly enough, “even this simple robot can be said to ‘experience' the present, ‘remember' the past, and also ‘feel' a flow of time” (Callender, 2017, p. 233).

Are Hartle and Callender claiming that such a robot with the right sensory apparatus, hardware, and software would be having such conscious experiences? In other words, are they literally claiming that such a robot is the answer to the hard problem of consciousness and the explanatory gap? I honestly cannot tell, although Callender does forego addressing the “mind/body problem” (p. 29) and suggests elsewhere that IGUS is a “toy model”, a proof of concept. If, however, they mean this literally, let me be the first to place a bet that such a robot would be experiencing nothing whatsoever. Build it and let us find out. Perhaps the more charitable interpretation is that once we figure out how brains or computational devices could be having any experiences at all, IGUS might explain why they are having these particular temporal experiences.

The claim here is that the explanation for the experience of Presence, Passage, and Direction (PPD) must lie with cognitive neuroscience, thus making PPD secondary properties, like color. There are several philosophers, physicists, and cognitive scientists who argue that the brain must somehow *generate* the experience of PPD. Here is an analogy. The brain is somehow like an old-school movie projector that takes a static series of still frames (the block universe) and creates the “illusion” of PPD. However, instead of a film projector, we have IGUS. But one needs only contemplate this idea for a second or two to see the problem. Barring radical emergence, if physics is “frozen” in the block universe, then so are brains. The brain (i.e., the static 4D worldline of a brain in spacetime) cannot be the analog of the film projector, because it states no more movement or flow than anything else in spacetime. The “activities” of the brain are just more events “frozen” in the still frames; therefore, the brain is not like the film projector that brings PPD to the game “from the outside”. Falling back on the “dynamical activity” of the brain poses the question of how a brain in a block universe could *generate, produce*, or *cause* **any** conscious experience, especially those involving PPD.

IGUS might get a pass on the hard problem (though of course, temporal experience is just a central subset of that problem), but it still must explain *the contents* of phenomenal consciousness, for example, as it pertains to temporal experience. Here, the same issue looms again. A brain “running” the IGUS program in a world with no objective Passage, Presence, and Direction, in a world with nothing but Humean regularities relating to 4D snapshots, is just a succession, a continuum of snapshots with a certain causal or temporal ordering. Such brain states “implementing” IGUS are *merely correlated* with a conscious precept on each slice or slices of that brain's worldtube. Those brains cannot *produce* or *structure* phenomenal consciousness more or less actively than physics can in such a world. It is IGUS *conceived as a dynamical computational process* that is supposed to explain what the physics in such a world cannot, but it has no more resources to do this causally or dynamically than the physics itself.

Take again the analogy of a film reel and projector—a very simple IGUS. Buonomano expresses this view perfectly with his claim that “the brain is a time machine” that produces the subjective experience of Passage and Presence (Buonomano, 2017). It is the movement of the film strip through the projection that yields the temporal experience. But, in the block universe, what plays the role of the projector? From a “God's-eye” perspective, nothing is moving; there is nothing to play the role of the projector. Brain states are no better off in this regard than any other physical process. As Dainton (2001, p. 389) states, “it is a mistake to conclude…that a continuous stream of consciousness can be formed merely by placing momentary experiences with static contents side-by-side, as it were...there is all the difference in the world between watching a movie and looking at a collection of still images”. In such a world, it is very hard to see how brains could be “time machines” or “producers” of conscious content in any sense.

My conclusion is that neither physics nor cognitive neuroscience has time for the other. To see another way to fix this problem of time, take the time to read our book. We argue that time is neither a projection of the brain, nor is it built into fundamental physics. Time is a relational property of embodied agents, not a secondary property projected by brains. Indeed, our claim is that the primary/secondary distinction is a bad one. At least for basic Passage, Presence, and Direction, we seek to erase the dualism between the mind's time and the world's time. Thus, we defend a Jamesian brand of neutral monism which holds that the mental and the physical are neutral and non-dual–there is just one thing. In this view, physics begins and ends with experience. “Physics” is best conceived as constraints on what embodied experiences are possible, for example, the light postulates and relativity principle of special relativity. Thus, the “ant's-eye” view with Passage, Presence, and Direction, is just as real and fundamental as the “God's-eye” perspective of eternalism.

From the perspective of neutral monism, the claim that the world is carved at the joints in terms of physical/mental, inner/outer, subject/object, etc., is not a datum, but rather an inductive projection. As James (1905a,b, p. 1208) puts it, “Subjectivity and objectivity are affairs not of what an experience is aboriginally made of, but of its classification”. Allegedly “inner” experience is not inherently or essentially mental, and the so-called “external” world isn't inherently non-mental or physical. “Pure experience” (as James calls it), in itself, “is no more inner than outer…. It becomes inner by belonging to an inner, it becomes outer by belonging to an outer, world” (p. 217). As James scholars have often noted, his “views were not well received or accurately interpreted” in his own time (p. xi). Some have even portrayed James' view as a kind of eliminativism or behaviorism because he says things of this nature, “Consciousness, as it is ordinarily understood, does not exist” (p. 109). James isn't denying the existence of conscious experience as such, but only a particular conception of consciousness, namely he is rejecting the idea of consciousness as qualia (inner tropes of experience that could exist without a subject as something over and above subjectivity). People often fail to appreciate this point because they leave out the second half of the preceding quote, “any more than does matter” (p. 109). Taking the quote in full, we see that James is really rejecting the primary/secondary property distinction and the idea that matter is a substance with essentially, intrinsic physical properties. Unlike panpsychism, James is not replacing intrinsic physical properties with essentially qualitative ones such as qualia or subjectivity.

As Thompson (2015, p. 61) notes in what follows, the view James espouses under the name neutral monism or radical empiricism has much more ancient roots in Buddhism and perhaps Hinduism:

Take a moment of visual awareness such as seeing the blue sky on a crisp fall day. The ego consciousness makes the visual awareness feel as if it's “my” awareness and makes the blue sky seem[sic] the[sic] separate and independent object of “my” awareness. In this way, the ego consciousness projects a subject–object structure onto awareness. According to the Yogacara philosophers, however, the blue sky is not a separate and independent object that is cognized by a separate and independent subject. Rather, there is one “impression” or “manifestation” that has two sides or aspects—the outer-seeming aspect of the blue sky and the inner-seeming aspect of the visual awareness. What the ego consciousness does is to reify these two interdependent aspects into a separate subject and a separate object, but this is a cognitive distortion that falsifies the authentic character of the impression or manifestation as a phenomenal event.

Per neutral monism, there is no PPD without a subject/object cut (subjectivity), which requires some sort of embodiment. As Taylor (1996, p. xii) put it, “James' metaphysics of pure experience is aimed directly at the dualisms of mind and body and knower and known (subject and object, thought and thing, representation and represented, and consciousness and content)”. There is no subject without an object and vice versa. It is this cognitive “cut” that leads to the experience of an ontologically distinct agent in a world in space and time.

Callender (2017, p. 262) notes that the IGUS temporal structure is contingent, and we can imagine radically different temporal structures consistent with the laws of physics and M4. In very interesting work, Gruber et al. (2020; 2022, using virtual reality (VR), instantiates some of these alternatives. However, that one can induce such changes to temporal experience should surprise no one and certainly does not confirm the IGUS account per se, or the idea that PPD is a projection of the brain. The alternative temporal worlds are imposed by VR on subjects who are already experiencing PPD. Thus, such experiments do not resolve the concerns I raised about accounting for Passage, Presence, and Direction in a block universe with just IGUS. Through experimental and pharmacological means (such as visual or bodily illusions and psychedelics), one can induce radically different alternative experiences in people regarding all sorts of perceptions, etc., but it does not follow that every experience is merely a secondary property projected by the brain. All experience is contingent in the sense that it can be radically altered without altering the physics of time as such. This does not mean that say the metric in M4 is not partly responsible for our everyday experience of time.

I think these experiments make the point that the phenomenology of time is relational. From the perspective of neutral monism, what such alternative VR worlds do is de facto “change the physics.” For example, a VR world or full-blown simulation with closed-time-like curves is the equivalent of living in an “actual” world with closed-time-like curves. We could be living in a simulation now. Exactly what temporal experiences are possible or not given certain constraints is of course an open and interesting question.

## Author contributions

The author confirms being the sole contributor of this work and has approved it for publication.
